# A systematic review of the psychometric properties of the Boston Carpal Tunnel Questionnaire

**DOI:** 10.1186/1471-2474-7-78

**Published:** 2006-10-20

**Authors:** Jose C de Carvalho Leite, Christina Jerosch-Herold, Fujian Song

**Affiliations:** 1 School of Allied Health Professions, University of East Anglia, Norwich, UK

## Abstract

**Background:**

The Boston Carpal Tunnel Questionnaire (BCTQ) is a disease-specific measure of self-reported symptom severity and functional status. It is frequently used in the reporting of outcomes from trials into interventions for carpal tunnel syndrome. We conducted a systematic review of published studies on the psychometric properties of the BCTQ to determine the level of evidence on the instrument's validity, reliability and responsiveness to date.

**Methods:**

A search of the databases Medline, CINAHL, AMED and PsychInfo was conducted to retrieve studies which investigated one or more of the psychometric properties of the BCTQ. Data abstraction was undertaken by the first two authors.

**Results:**

Ten studies were retrieved which met the inclusion criteria. One study evaluated face and content validity (43 patients) eight studies assessed construct validity (932 patients), four studies tested reliability (126 patients) and nine studies assessed responsiveness (986 patients). Interpretability was evaluated in one study and acceptability in eight studies (978 patients).

**Conclusion:**

The BCTQ is a standardised, patient-based outcome measure of symptom severity and functional status in patients with carpal tunnel syndrome. The evidence base of the psychometric properties indicates that the BCTQ is a valid, reliable, responsive and acceptable instrument and should be included as a primary outcome measures in future CTS trials.

## Background

The effectiveness of surgical and non-surgical interventions for carpal tunnel syndrome (CTS) has been investigated through randomised controlled trials and systematic reviews [1-4]. The outcomes reported in trials of interventions for CTS are wide ranging and include relief of symptoms, time to resumption of activities and work, clinical measures of sensation, strength and dexterity, electrophysiological studies and the use of validated patient-based questionnaires of symptom relief, functional ability and health-related quality of life and satisfaction. The use of patient-based outcome measures is becoming more wide-spread and reflects the need to incorporate patient perspectives on outcome[5]. Some patient-based questionnaires are region-specific, that is, they are applicable to the upper limb only, e.g. the Patient Evaluation Measure (PEM) [6] and Disabilities of the Arm, Shoulder and Hand (DASH) [7] whilst others are generic measures of quality of life, for example, the Medical Outcomes Short-Form-36 (SF-36)[8]. In order to encompass all relevant outcomes a combination of generic and specific measures need to be employed [9].

The Boston Carpal Tunnel Questionnaire (BCTQ), also referred to as the Levine scale[10], Brigham and Womens' Carpal Tunnel Questionnaire [11] and Carpal Tunnel Syndrome Instrument [12], is a patient-based outcome measure that has been developed specifically for patients with CTS. It has two distinct scales, the Symptom Severity Scale (SSS) which has 11 questions and uses a five-point rating scale and the Functional Status Scale (FSS) containing 8 items which have to be rated for degree of difficulty on a five-point scale. Each scale generates a final score (sum of individual scores divided by number of items) which ranges from 1 to 5, with a higher score indicating greater disability. The BCTQ has been used as an outcome measure in clinical studies, and has also undergone extensive testing for validity, reliability and responsiveness. The purpose of this paper is to review and synthesise the evidence on the psychometric properties of the BCTQ published to date, and to make recommendations regarding its use in practice and research.

## Methods

### Search strategy and review criteria

The review considered all studies designed primarily to investigate an aspect of validity, reliability or responsiveness of the BCTQ in patients with carpal tunnel syndrome. We also considered any studies reporting on interpretability and patient acceptability of the BCTQ.

The bibliographic databases Medline (1966–2005), CINAHL (1982–2005), AMED (1985–2005) and PsycINFO (1887–2005) were searched using the following MeSH terms: carpal tunnel syndrome, outcome assessment, questionnaires, psychometrics, validity, reliability, reproducibility, responsiveness. Keyword searches were also made using 'Boston Carpal Tunnel Questionnaire' and 'carpal tunnel instrument'. Bibliographies of the articles obtained were checked to identify any studies not retrieved through the electronic databases. The search was limited to English language articles only.

The title and abstract of the articles retrieved were read and selected for inclusion if they fulfilled the following criteria: a prospective, observational study or clinical trial designed to evaluate validity, reliability, responsiveness, acceptability or interpretability of the BCTQ in patients with CTS. The full text was obtained for those articles which met the inclusion criteria.

A data extraction form was developed and used to summarise information regarding the psychometric properties assessed including design, methods, sample and main results of those studies included (see Additional file 1). Each article was independently read by the first two authors and a data extraction form completed. Any discrepancies between reviewers were discussed and agreed. The data from the studies were summarised in tables, and then qualitatively synthesized.

### Psychometric properties assessed

The psychometric properties of outcome measures should be assessed by their face and content validity, construct validity, inter-tester and intra-tester reliability, responsiveness, interpretability, and acceptability and responder burden[13]. A full explanation of these concepts is beyond the scope of this paper and the reader is referred to Fitzpatrick et al [13]or Norman and Streiner [14], however a brief definition of these psychometric criteria in the context of patient-based questionnaires is given in Additional file 2.

## Results

The search yielded 21 hits. After reading the titles and abstracts, eleven studies were excluded because they did not include the BCTQ (n = 7); the BCTQ was used as a criterion measure for other instruments (n = 1); the BCTQ was applied as a measure to ascertain the incidence or severity of CTS (n = 2) or the BCTQ was compared against other diagnostic tests designed to detect CTS (n = 1).

A total of ten studies which were primarily designed to evaluate one or several psychometric properties of the BCTQ were included [10-12,15-21]. All these studies applied the questionnaire in adults (aged 18 to 90 years old) with a diagnosis of carpal tunnel syndrome (Table 1).

Face and content validity were examined in a study which led to the original development of the BCTQ [10] construct validity was assessed in eight studies, totalling 932 patients; responsiveness in nine studies (986 patients); test-retest reliability in four studies (126 patients); acceptability in eight studies (978 patients); and interpretability in one study (196 patients) (Table 1). A cross-sectional design was used in studies assessing face and content validity [10] and interpretability of the BCTQ [16]. The four studies which investigated reliability and the nine studies assessing responsiveness used prospective cohort data. One study [10] also used retrospective cohort data to assess responsiveness which has not been reported here due to its limited value. The study design was observational for almost all studies, except one [19] which used data from a randomized controlled trial. Study power was considered in three out of nine studies. In both the studies by Bessette et al [16] and Katz et al [18] patients were recruited through a community-based observational study in Maine between 1992–93 and therefore it is possible that the same patients were included in both datasets.

### Face and content validity

Only one study evaluated face and content validity of the BCTQ [10]. The results suggest that the two sub-scales of the questionnaire measure severity of symptoms and functional status, which are considered the most important reasons for seeking treatment. The Functional Status Scale covers activities usually performed by a broad range of patients, but does not include items relevant to specific groups such as workers.

### Construct validity

The BCTQ was compared with 12 other outcome measures for carpal tunnel syndrome to assess construct validity (see Table 2). The hypothesized relationships between the BCTQ and the other outcome measures were assessed by Spearman rank correlation coefficients [10-12], [15-18] and by one-way analysis of variance to assess the extent to which postoperative symptom severity and functional status were related to patient satisfaction[12]. All observed correlations were in the expected direction. There were high correlations between the BCTQ and the Disabilities of the Arm, Shoulder and Hand Questionnaire (DASH)[11] (r = 0.90 and r = 0.87, p < 0.001), and between the BCTQ and the Arthritis Impact Measurement Scale-2 (AIMS-2) (r = 0.71 p < 0.01)[15]. Moderate correlations were found between the BCTQ and measures of symptom relief, generic measures of health status, quality of life and satisfaction (r-values ranged from 0.50 to 0.56). The analysis of the relationship between patient satisfaction with the overall results of surgery and the BCTQ symptom severity and Functional Status Scales showed worse scores for both scales (p < 0.001) in patients with lower degree of satisfaction.

The association between the BCTQ and score from clinical sensory tests was weak (r = 0.15 to 0.17 for Symptom Severity Scale, and r = 0.24 to 0.42 for functional status score). The correlations between pinch and grip strength and the two subscales of the BCTQ were moderate with higher values for the Functional Status Scale than the Symptom Severity Scale, whereas sensibility measures showed a stronger association with the Symptom Severity Scale (Table 2).

Internal consistency was assessed by correlating all the scores from the individual items on the BCTQ with the overall score on the BCTQ. The Cronbach alpha values ranged from α = 0.80 to 0.90 for the symptom severity scale and from α = 0.88 to 0.93 for the Functional Status Scale. A *known-groups *validation method was applied in one study. Katz et al [18]compared satisfaction with change in functional status and in symptom severity (BCTQ), perceived improvement in quality of life and perceived improvement in symptoms severity between recipients and non-recipients of workers' compensation. As hypothesized, there was evidence of a difference between the two groups of patients for the BCTQ Functional Status Scale and the Symptom Severity Scale (Fisher's Z transformation applied to Spearman coefficient, p < 0.05).

### Reliability of the BCTQ

Test-retest reliability was reported in four studies. Levine et al [10] assessed reliability by administering the questionnaire on two successive days. Pearson's correlation coefficients showed high correlation between the scores (r = 0.91 and 0.93 for symptom severity and Functional Status Scales, respectively). Greenslade et al[21] assessed reliability by applying the BCTQ in a two-weekly interval in patients awaiting surgery. Test-retest plots, difference between means and Pearson's correlation coefficients were the measures reported. The mean differences between test and retest scores (*Δ*) were not significantly different from zero, and correlations were high (for the Symptom Severity Scale *Δ *= 0.1, 95% CI = -0.1 to 0.3, r = 0.82; for the Functional Status Scale *Δ *= 0, 95% CI = -0.2 to 0.2, r = 0.79). In a third study [12], the BCTQ was applied two times before surgery with a mean interval of 14 days. Reliability was measured by difference between means and Spearman correlation coefficients. The mean differences between test and retest scores (*Δ*) were not significantly different from zero, and correlations were moderate (for the Symptom Severity Scale *Δ *= -0.1, p < 0.05, r = 0.64; for the Functional Status Scale *Δ *= 0.08, p < 0.05, r = 0.71). Test-retest reliability was assessed of the Spanish version of the BCTQ in a prospective study of 42 patients with confirmed CTS. Pearson correlation coefficients were reported as r = 0.87 for the Symptom Severity Scale and r = 0.85 for the Functional Status Scale [20].

### Responsiveness of the BCTQ

Responsiveness was assessed in prospective follow-up studies of surgical interventions only and reported as effect size (ES) or standard response mean (SRM) (Table 3). The time interval used to calculate change ranged from 1 1/2 to 6 months post-operatively with responsiveness indices increasing in magnitude through larger time intervals. Two studies assessed responsiveness of the BCTQ based only on patients reporting greater satisfaction with the results of surgery [16,19]. Eight studies reported responsiveness indices separately for each BCTQ subscale. The effect sizes for the Functional Status Scale ranged from 0.48 at 6 weeks to 1.44 at 27 weeks post-surgery, and for the Symptom Severity Scale it ranged from 1.13 at 13.5 weeks to 2.33 at 27 weeks post-surgery. Effect sizes and SRMs for the Symptom Severity Scale tended to be higher than for the Functional Status Scale, however both scales yielded moderate (>0.5) to large (>0.8) responsiveness indices supporting the notion that both scales are sensitive to clinical change in patients undergoing surgical interventions. The use of Effect sizes as responsiveness indices tended to generate slightly larger values than when the SRM was used, hence they should not be compared directly. The responsiveness of the BCTQ total score was reported in one prospective cohort study [16] (not in Table 3). This study assessed the relative responsiveness to change of generic versus disease-specific as well as unweighted versus weighted health status measures in carpal tunnel syndrome. The weighted disease-specific health status measure was obtained by asking the subjects how important relief of the specific symptom or improvement of the specific function measured by the BCTQ was to the decision to have surgery. The weighted-BCTQ score (SRM = 1.56, ES = 1.99) was more responsive than the unweighted score (SRM = 1.36, ES = 1.57). The generic health status measures were less sensitive to change than the BCTQ.

### Acceptability of the BCTQ

Acceptability was examined in eight studies (Table 1). The burden of completing the BCTQ was reported as minimal in two studies [10,15] based on no loss to follow-up. Greenslade et al[21] reported the mean time taken to complete the BCTQ as 5.6 minutes (± 3.5 min). Loss to follow-up or incomplete responses ranging from 1% to 10% were observed in four of the eight studies [12,16,17,21] and reached 19% in two studies[11,18]. Bessette et al [16] reported that only nine out of 231 subjects who completed the 6 month follow-up evaluation did not complete the BCTQ, giving a response rate of 96%. Greenslade et al [21]showed that two out of 312 pre- and post-operative questionnaires returned had missing information in the Symptom Severity Scale and 17 out of 312 in the functional status scale, which corresponds to a response rate of 99% and 95% respectively. There are no recommendations in the literature to date with regards to how missing responses should be managed and what the threshold for number of incomplete items is which would render the subscale data invalid.

### Interpretability of the BCTQ

Interpretability was assessed in a study including 196 subjects[16]. Using the satisfaction with the outcomes of surgery as a discrete variable (unsatisfied, somewhat satisfied, and very or completely satisfied), the minimal clinically important difference (MCID) was estimated as the mean difference between the BCTQ scores before surgery and at 6 months after surgery for the unsatisfied and somewhat satisfied patients. The MCID is 0.74 for the BCTQ (total score based on the average of both subscales with scale ranges from 1 to 5), a value considered superior to generic measures, e.g. SF-36, in distinguishing clinically important differences after carpal tunnel release. The MCID for individual scales has not been reported, however Atroshi et al [12] also presented summary statistics for each subscale according to those patients who were satisfied, somewhat satisfied and dissatisfied. The mean change between pre- and postoperative scores for the Symptom Severity Scale for the satisfied, somewhat satisfied and dissatisfied patients were 1.6, 1.0 and 0.2 respectively, indicating that a minimum difference of 0.8 can be deemed as clinically important using patients satisfaction as a criterion. For the Functional Status Scale the mean change pre- and post-operatively were 1.0, 0.6 and 0.1 for the satisfied, somewhat satisfied and dissatisfied patients respectively, suggesting that a value of 0.5 is clinically important.

## Discussion

The ten selected studies presented some strength. The studies sampled a wide age range of participants, which is desirable considering that items of relevance for the young and the elderly are incorporated [13,22]. Sample sizes appeared to be adequate to yield stable correlations, although power calculations were reported in three studies only. The studies used prospective cohort data to assess the majority of the BCTQ psychometric properties. Prospective cohort studies are at greater risk of missing data during the follow-up phase which in turn can lead to systematic measurement error. However this does not appear to be a major problem in these studies, since participation rates were relatively high with losses of follow-up varying from none to 19%.

Limitations must also be acknowledged. Firstly, none of the ten studies assessed all the psychometric properties, making comparisons difficult specially regarding face and content validity, acceptability, interpretability and reliability. Secondly, the factor structure of the BCTQ, an aspect of construct validity, has not been examined in the selected studies. Factor analysis is a method of assessing the construct validity of a questionnaire. In confirmatory factor analysis, the scores from each item in the scale would show high loadings, expressed as high Eigenvalues, on one of the predicted factors (e.g., symptom severity and functional status of the BCTQ). It has been hypothesized that the BCTQ comprises a two-factor structure consistent with symptom severity and functional status [10]. Because the constructs assessed are so distinct (symptom severity and functional status), it is likely that the BCTQ total score is less informative and helpful for clinical purposes even though four studies reported the total score. Also, Katz et al[18] found the two-factor structure consistent with the symptom severity and factor structure scales in workers' compensation recipients, however this large study did not investigate the BCTQ factor structure; either confirming the original structure or suggesting an alternative factor models may provide a better explanation of that data. Thirdly, test-retest reliability was reported in four studies and Pearson correlation coefficient was used in two of them[10,20], a statistical approach recognized as inappropriate as it only measures the strength of association between scores and not agreement[23]. Forthly, in one study [10] the analyses of responsiveness of the BCTQ compared data from two independent cohorts (one prospective and another retrospective). It is likely that the information obtained retrospectively is less accurate than the prospective one.

Validity of the BCTQ was assessed in terms of face, content and construct validity. Face and content validity were assessed through consultation with individuals with relevant expertise in order to generate the content of the questionnaire. The content of the BCTQ had been examined in one study[10], suggesting that the questionnaire items match the test objectives and the impact of carpal tunnel syndrome on patients' daily life. The construct validity of the BCTQ had been assessed in the majority of the ten studies. In the selected studies construct validity it was assessed as the extent to which the items of the BCTQ 'behaved' the way that the construct it purports to measure (that is symptom severity and functional status) should 'behave' with regard to other established measures (e.g., the Disabilities of the Arm, Shoulder, and Hand Questionnaire, pinch and grip strength, satisfaction with the outcomes of surgery). Stronger correlations were observed between the BCTQ and the other disease- and region-specific measures such as the DASH and Arthritis Impact Measurement Scale, than between the BCTQ and generic objective measures such as SF-36 and Quality of Life Questionnaire indicating greater overlap between the former measures. The BCTQ also demonstrates construct validity when its internal consistency was examined. A high Cronbach alpha indicates homogeneity of items and supports the validity of the construct being tested [14].

Responsiveness to clinical change is another important feature of an outcome measure. The data on effect sizes and standard response means demonstrated that the Symptom Severity Scale and Functional Status Scales are able to detect clinically meaningful change resulting from the treatment for carpal tunnel syndrome and yielded large effect sizes over a 6 month interval. However in two of the studies the data on responsiveness were based on a subgroup of patients reporting greater satisfaction with surgery. Responsiveness indices in these are therefore likely to be larger than in the other studies. Using a responsive outcome measure will facilitate the detection of moderate treatment effects in clinical research.

The BCTQ has shown good levels of acceptability with response rates of 90% and above and takes less than 10 minutes to complete. The interpretability has been assessed in relation to patient satisfaction with the outcomes of surgery and an overall difference of 0.74 has been designated as the minimally clinical important difference.

This review considered English language publications only, however there are a number of published studies which have used translations of the BCTQ into other languages including Italian [24] Swedish [12] Portuguese [25] and Spanish [20] widening the applicability of the BCTQ to non-English speaking settings.

Scale development is an ongoing process which may never be complete. The properties such as the validity, reliability and responsiveness investigated to date are not fixed properties but specific to the instrument used in a given situation and with a given population. The BCTQ was developed for use in heterogeneous samples of patients of a wide age range with CTS. Further research is needed to examine the consistency of its psychometric properties, with special attention to the factor structure, among specific populations, test-retest reliability using appropriate statistical measures and defining the MCID for each subscale against appropriate external criteria.

Clinicians looking for a disease-specific measure for assessing pre- and post-operative symptom severity and functional status can be confident that the BCTQ is responsive to change, repeatable over time and that the scales measure what they purport to measure. The BCTQ is also acceptable and quick to administer and as it relies on self-report can be applied via postal methods.

## Conclusion

In summary, the BCTQ offers a standardised patient-based outcome measure of symptom severity and functional status for which there is good evidence on validity, reliability and responsiveness and it should be recommended for inclusion in future trials on carpal tunnel interventions.

## Competing interests

The author(s) declare that they have no competing interests.

## Authors' contributions

JCL carried out the searching and reviewing of studies and drafted the manuscript. CJH conceived of the original idea, obtained funding and participated in the search, review and drafting of the manuscript. FS provided expert advice on systematic reviewing and contributed to the manuscript. All authors read and approved the final manuscript.

## Pre-publication history

The pre-publication history for this paper can be accessed here:



## Supplementary Material

Additional file 1 Data extraction form (word document) Click here for file

Additional file 2 Definitions of psychometric properties (word document) Click here for file

## Figures and Tables

**Table 1:** Summary of studies assessing the psychometric properties of the Boston Carpal Tunnel Questionnaire (BCTQ)

**Studies**	**psychometric properties assessed**						**Methods**				
	
	Face/Content validity	Construct validity	Responsiveness	Reliability	Acceptability	Interpretability	Population	Design*	Observational (O) RCT	Study Power	No. of Patients included in the analyses
Amadio et al, 1996	.	√	√	.	√		33–80 years, 72% women	P	O	Yes	n = 22
Atroshi et al, 1998	.	√	√	√	√		21–88 years 66% women	P	O	No	Responsiveness/Construct validity n = 102 Test-retest n = 22
Bessette et al, 1998	.	√	√	.	√	√	> 18 years, 84% women	P/C	O	No	n = 196
Gay et al, 2003	.	√	√	.	√		31–81 years, 55% women	P	O	Yes	n = 40
Greenslade et al, 2004	.	.	√	√	√		> 49 years, 72% women	P/C	O	No	Acceptability = 88 Responsiveness n = 57 Test-retest n = 31
Katz et al, 1994	.	.	√	. .	.		> 18 years, 70% women	P	RCT	Yes	n = 43
Katz et al, 1996	.	√	√	.	√		18–55 years, 70% women	P	O	No	n = 268^§^
Levine et al, 1993	√	√	√	√	√		19–88 years, 75% women	P/R/C	O	No	Construct validity n = 43 Responsiveness n = 39 Test-retest n = 31
Mondelli et al, 2002	.	√	√	.	√		20–90 years, 81% women	P	O	No	n = 219
Rosales et al, 2002		√		√			34–63 years 86% women	P	O	No	N = 42

Legend:

* P = Prospective, R=Retrospective (R), C = Cross-sectional

^§ ^Workers compensation recipients n = 113, non-recipients n = 155

**Table 2:** Comparative measures used in studies assessing the construct validity of the BCTQ

**Comparative Measures**	**BCTQ construct validity (Spearman rank correlations)**		
	
	**BCTQ – total score**	**Symptom Severity Score**	**Functional Status Score**
**Symptoms**			
Historical-objective severity scale	Significant correlation (p < 0.001) (Mondelli et al, 2002)		
Duration of symptoms	Significant correlation (p < 0.001) (Mondelli et al, 2002)		
Expectation of symptom relief	0.51 (.56^§^) (Bessette et al, 1998)		
Symptom relief	0.51 (0.56^§^) (Bessette et al, 1998)	0.59 (Katz et al, 1996)* 0.31 (Katz et al, 1996)**	0.48 (Katz et al, 1996)* 0.19 BCTQ (Katz et al, 1996)**
**Nerve conduction studies**			
Median-nerve sensory conduction velocity		0.11 (Levine et al, 1993)	0.12 (Levine et al, 1993)
Electrophysiological study	Significant correlation (p < 0.001) (Mondelli et al, 2002)		
**Clinical sensory tests**			
Semmes-Weinstein monofilaments		0.17 (Levine et al, 1993)	0.24 (Levine et al, 1993)
2-point discrimination		0.15 (Levine et al, 1993)	0.42 (Levine et al, 1993)
**Clinical motor tests**			
Pinch strength	0.73 (Amadio et al, 1996)	0.47 (Levine et al, 1993)	0.60 (Levine et al, 1993)
Grip strength	0.87 (Amadio et al, 1996)	0.38 (Levine et al, 1993)	0.50 (Levine et al, 1993)
**Patient-based measures of function**			
DASH (6 wk post-operative)	0.90 (Gay et al, 2003)		
DASH (12 wk pos-toperative)	0.87 (Gay et al, 2003)		
Arthritis Impact Measurement Scale	0.71 (Amadio et al, 1996)		
**General Health Status, Quality of life and satisfaction**			
36-item Short-form Health Survey	0.70 (Physical role scale) (Amadio et al, 1996)	0.20 – 0.64 ^‡ ^(Preoperative) (Atroshi et al, 1998) 0.33 – 0.64 ^‡ ^(Postoperative) (Atroshi et al, 1998)	0.29 – 0.70 ^‡ ^(Preoperative) (Atroshi et al, 1998) 0.34 – 0.67 ^‡ ^(Postoperative) (Atroshi et al, 1998)
Quality of life	0.50 (0.57^§^) (Bessette et al, 1998)	0.68 * (Katz et al, 1996) 0.37 ** (Katz et al, 1996)	0.54 * (Katz et al, 1996) 0.41 ** (Katz et al, 1996)
Satisfaction with the outcomes of surgery	0.56 (0.62^§^) (Bessette et al, 1998)	0.69 * (Katz et al, 1996) 0.37 ** (Katz et al, 1996)	0.55 * (Katz et al, 1996) 0.21 ** (Katz et al, 1996)

**Legend**:

† – Spearman's rank correlation coefficient and nonparametric test used due to non-normal distribution of the outcome scales.

§-Weighted disease-specific health status measure in BCTQ (For each item subjects were asked how important relief of the specific "symptom" or improvement of the specific "function" was to the decision to have surgery.)

‡ – Spearman correlation coefficient (minimum and maximum) between the BCTQ overall score and the 8 scales of the 36-item Short-form Health Survey.

* – Workers' compensation recipients

** – Workers' compensation non-recipients

**Table 3:** Responsiveness Indices for the BCTQ Symptom Severity and Functional Status Scales

**Study**	**Responsiveness Indices (Measure of Clinical Change)**		
	
	**BCTQ Functional Status Score (FS)**	**BCTQ Severity of Symptoms Score (SS)**	**Assessment interval**
Amadio et al, 1996	SRM = 1.26	SRM = 1.75	Before and 13.5 weeks post surgery (n = 22)
Atroshi et al, 1998	SRM = 0.94 (0.72–1.16) ES = 0.94	SRM = 1.7 (1.4–2.0) ES = 2.1	Before and 13.5 weeks post surgery (n = 102)
Gay et al, 2003	At 6 weeks after surgery SRM = 0.46; ES = 0.48 At 12 weeks after surgery SRM = 1.05; ES = 1.05	At 6 weeks after surgery SRM = 1.67; ES = 1.74 At 12 weeks after surgery SRM = 2.01; ES = 1.96	before, 6 and 12 weeks post surgery (n = 34)
Greenslade et al, 2004	SRM = 0.62	SRM = 1.07	before and 13.5 weeks post surgery (n = 57)
Katz et al, 1994	SRM = 0.96 ES = 0.86	SRM = 2.02 ES = 2.03	before and 13.5 weeks post surgery (n = 43)
Katz et al, 1996*	Male WC/WCNon ES = 0.65/ES = 0.76 Female WC/WCNon ES = 1.25/ES = 1.44	Male WC/WCNon ES = 1.43/ES = 1.33 Female WC/WCNon ES = 1.63/ES = 2.13	before and 27 weeks post surgery (n = 268)
Levine et al, 1993	SRM = 0.71† greater satisfaction with the outcomes of surgery *versus *improvement of FS = 0.50 (p < 0.01)	SRM = 1.13† greater satisfaction with the outcomes of surgery *versus *improvement of SS = 0.54 (p < 0.01)	before and 13.5 weeks after surgery (n = 26)
Mondelli et al, 2002	ES = 1.23§	ES = 2.33§	before and 27 weeks post surgery (n = 219)

Legend:

WC – Workers' compensation recipients

WCNon – Workers' compensation Nonrecipients

* ES were not reported in paper but have been calculated based on values given in tables

† responsiveness reported as ES, however calculation given is SRM (mean change/S.D change)

§responsiveness assessed as differences but ES calculated from values given in paper
