# Supplementary material for: A systematic review of the psychometric properties of the Boston Carpal Tunnel Questionnaire
Source: BMC Musculoskelet Disord. 2006 Oct 20;7:78. doi: 10.1186/1471-2474-7-78 (PMC1624826; doi:10.1186/1471-2474-7-78)
Supplement: Additional file 1 — Data extraction form (word document) [file 1471-2474-7-78-S1.doc]

| Date: | | | | Reviewer: | | | | |
| --- | --- | --- | --- | --- | --- | --- | --- | --- |
| Journal: | | | | | | | | Country: |
| Title: | | | | | | | | |
| Instrument name: | | | | | | | | |
| Aim/Objectives: | | | | | | | | |
| Outcome measure(s) being evaluated: | | | | | | | | |
| Domains: | | | | | | Equipment/instrument: | | |
| Standardised protocol described or referenced: ( ) | | | | | | | | |
| How was diagnosis confirmed? | | | | | | | | |
| **Face / Content validity** Clinical expert consensus ( ) patient input ( ) | | | | | | | | |
| **Construct validity** No info ( )  Convergent yes( ) no( )  Comparative measure:  Sample: | | | Internal consistency ( ) | | | | PC/factor analysis ( ) | |
|  |  |  |  |  |  |  | Results: | |
| **Inter-tester Reliability**  No info ( ) | Results: | | | | | | | |
| Sample: | | Methods: | | | | | | |
| **test-retest Reliability**  No info ( ) | Results: | | | | | | | |
| Sample: | | Methods: | | | | | | |
| **Responsiveness**  No info ( ) | Results: | | | | | | | |
| Sample: | | Methods: | | | | | | |
| **Scaling and response categories:** | | | | | | | | |
| **Interpretability:** MCID defined ( ) normative data ( ) | | | | | | | | |
| **Acceptability** *(respondent burden/distress, missing responses, refusal rates)*: No info ( ) | | | | | | | | |
| **Time to complete:** No info ( ) | | | | | **Shorter forms available:** yes( ) no( ) | | | |
| **Conclusions by authors**: | | | | | | | | |
| **Comments**: | | | | | | | | |
