# Supplementary material for: A systematic review of the psychometric properties of the Boston Carpal Tunnel Questionnaire
Source: BMC Musculoskelet Disord. 2006 Oct 20;7:78. doi: 10.1186/1471-2474-7-78 (PMC1624826; doi:10.1186/1471-2474-7-78)
Supplement: Additional file 2 — Definitions of psychometric properties (word document) [file 1471-2474-7-78-S2.doc]

**Definitions of psychometric properties**

Validity is the extent to which the questionnaire appears to measure what it purports to measure. Face and content validity pertain to how well the items of the questionnaire sample the domain of interest. Both can only be assessed through consultation with individuals with relevant expertise such as clinicians and patients in order to generate the content of the instrument. Construct validity is seen as a more rigorous form of validity as it can be assessed empirically. If a questionnaire is designed to assess a specific construct such as pain in CTS then it is postulated that it will also relate to other instruments which assess the same construct, for example by comparing it to another measure such as the verbal pain rating scale . Construct validity is often assessed quantitatively by examining the correlation coefficients between the instrument in question and other measures. A high correlation coefficient is indicative of convergent validity when there is evidence of similarity between measures of theoretically related constructs; and a low correlation coefficient is indicative of discriminant (or divergent) validity when the measures are of unrelated constructs [1] More complex methods of assessing construct validity include factor analysis and hypothesis testing [2]. Questionnaires are often made up of several questions or items or may contain subscales. Construct validity can also be established by examining the relationship between several underlying constructs or the inter-item correlations (assessed by Cronbach alpha). A high coefficient indicates good internal consistency or homogeneity of the items.

Reliability or reproducibility assesses the extent to which an instrument yields the same results on repeated applications [3]. Intra-tester reliability, also called test-retest reliability is often assessed by calculating a correlation coefficient, though this approach has been criticised as it may overestimate reproducibility and only measures the association between scores and not agreement. Intra-class correlation coefficients are more appropriate as they reflect both the degree of association and agreement between sets of scores[4]. A high reliability coefficient indicates good reproducibility, with coefficients of 0.7 or higher deemed adequate for research. Inter-tester reliability examines the stability of the instrument across several testers. This is not considered further here as it is not relevant in patient-based outcome measures.

Responsiveness can be defined as the degree to which a measure can detect change which is clinically meaningful[5]. It is assessed by measuring the magnitude of change in scores which occurs over time and as a result of an intervention, e.g. surgical release. There is no single agreed method of estimating responsiveness and a variety of statistical methods are advocated including effect size (calculated as the mean change divided by the standard deviation of the baseline score) and standard response mean (SRM) (mean change in score divided by standard deviation of change score). The larger the effect size or SRM the more sensitive the instrument is to clinically meaningful change. Values of 0.20, 0.50, and 0.80 or greater have been used to represent small, moderate, and large effect sizes respectively [6-8].

Interpretability relates to the meaning which can be derived from the numerical score obtained through the questionnaire. An approach which is increasingly used is to identify the ‘minimal clinically important difference’ (MCID). The MCID can be defined as ‘the smallest difference in score in the domain of interest which patients perceive as beneficial and which mandate, in the absence of troublesome side effects and excessive cost, a change in the patient’s management’ (page 408) [9]

A number of approaches have been used including anchor-based approaches linking change to other external criteria such as global ratings of change or preferences [10].

Acceptability is a further important criterion for evaluating patient-rated outcome measures as it relates to how likely to the patient is to complete it and therefore how good the response rates are. It can be assessed using criteria such as the time it takes to complete the questionnaire, the burden on the patient such as distress from answering difficult or sensitive questions and rates of missing responses or returns[3].

**References:**

1. deVellis RF: **Scale development: theory and applications**. London: Sage; 1991.

2. Portney L, Watkins M: **Foundations of clinical research: applications to practice**, 2nd edn. Upper Saddle River: Prentice Hall; 2000.

3. Fitzpatrick R, Davey C, Buxton MJ, Jones D: **Evaluating patient-based outcome measures for use in clinical trials**. In*.*: Health Technology Assessment; 1998: 1-74.

4. Bland J, Altman D: **Statistical methods for assessing agreement between two methods of clinical measurement**. *The Lancet* 1986, **1**(8476):307-310.

5. Liang M: **Evaluating measurement responsiveness**. *J Rheumatology* 1995, **22**:1191-1192.

6. Husted J, Cook R, Farewell V, Gladman D: **Methods for assessing responsiveness: a critical review and recommendations**. *Journal of Clinical Epidemiology* 2000, **53**:459-468.

7. Norman D, Streiner G: **Health Measurement Scales: a practical guide to their development and use**, 3rd edition edn. Oxford: Oxford University Press; 2003.

8. Stucki G, Liang MH, Fossel AH, Katz JN: **Relative responsiveness of condition-specific and generic health-status measures in degenerative lumbar spine stenosis**. *Journal of Clinical Epidemiology* 1995, **48**:1369-1378.

9. Jaeschke R, Singer J, Guyatt G: **Measurement of health status: ascertaining the minimal clinically important difference**. *Controlled Clinical Trials* 1989, **10**:407-415.

10. Crosby R, Kolotkin R, Williams G: **Defining clinically meaningful change in health-related quality of life**. *Journal of Clinical Epidemiology* 2003, **56**:395-407.
